# Pre-diagnostic plasma proteomics profile uncovers new biomarkers and mechanistic insights for incident kidney cancer

**DOI:** 10.1097/JS9.0000000000003474

**Published:** 2025-09-10

**Authors:** Wen Liu, Wei Chen, Dahai Dong, Guiming Zhang, Nianzeng Xing

**Affiliations:** a Department of Urology, National Cancer Center/National Clinical Research Center for Cancer/Cancer Hospital, Chinese Academy of Medical Sciences and Peking Union Medical College, Beijing, China; b State Key Laboratory of Molecular Oncology, National Cancer Center/National Clinical Research Center for Cancer/Cancer Hospital, Chinese Academy of Medical Sciences and Peking Union Medical College, Beijing, China; c Beijing Key Laboratory of Urologic Cancer Cell and Gene Therapy, National Cancer Center/National Clinical Research Center for Cancer/Cancer Hospital, Chinese Academy of Medical Sciences and Peking Union Medical College, Beijing, 100021, China.; d Department of Urology, The Affiliated Hospital of Qingdao University, Qingdao, China

**Keywords:** biomarkers, HAVCR1, kidney cancer, mediation analysis, proteomics, UK Biobank

## Abstract

**Background:**

The pathophysiological changes driving incident kidney cancer remain unclear. This study aimed to identify protein biomarkers and underlying mechanisms using pre-diagnostic plasma proteomics.

**Materials and methods:**

Among 48 851 UK Biobank participants, 165 were diagnosed with kidney cancer, and 2911 plasma proteins were analyzed. Dynamic changes in significant proteins were assessed up to 15 years before diagnosis using locally estimated scatterplot smoothing method. A mediation analysis using a four-component framework was conducted to evaluate the mediating role of proteomic features in the associations of body mass index (BMI) and smoking with kidney cancer risk. Additionally, an absolute shrinkage and selection operator regression model was developed for proteomics-based risk prediction.

**Results:**

Over a follow-up period exceeding 11 years, 24 proteins were significantly associated with kidney cancer risk (*P* < 0.05, Bonferroni-corrected for 2911), with Hepatitis A Virus Cellular Receptor 1 (HAVCR1) exhibiting the most statistically significant association (HR = 3.18, 95% CI: 2.70–3.74, *P* = 1.11 × 10^−40^). Trajectory modeling revealed that HAVCR1 exhibited the most significant fluctuations, with abnormal expression detectable up to 15 years before diagnosis. Unsupervised clustering identified four distinct protein trajectory patterns, suggesting different mechanisms may drive kidney cancer progression at various stages. Proteomic data mediated the effects of BMI and smoking on cancer risk, contributing 38.6% and 9.2% to the risk, respectively. The proteomic model significantly improved kidney cancer risk prediction compared to the clinical model (concordance index [C-index]: 0.811 vs. 0.713, *P* = 0.029), with HAVCR1 alone demonstrating comparable discriminative ability (C-index: 0.754).

**Conclusions:**

This large-scale plasma proteomics study highlights the potential of biomarkers, particularly HAVCR1, for early detection and insight into kidney cancer pathophysiology.

## Graphical abstract

**Figure:**
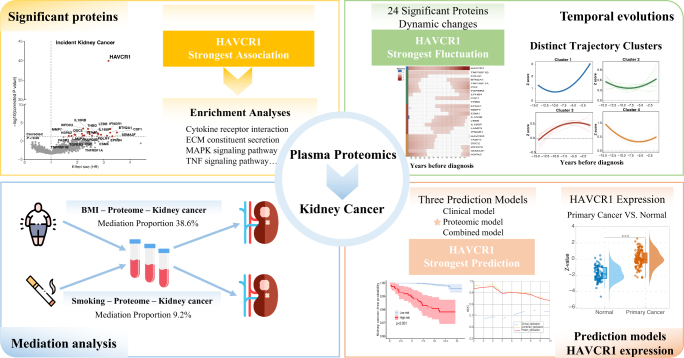

**HIGHLIGHTS**

- Plasma proteomics holds potential for predicting the onset of kidney cancer.
- HAVCR1 emerges as the plasma protein most strongly associated with kidney cancer, exhibiting significant pre-tumor alterations.
- Plasma proteins linked to kidney cancer display distinct alteration patterns, reflecting the pathological changes throughout the disease trajectory.
- Proteomics mediates the interactions between obesity, smoking, and kidney cancer risk.

## Introduction

Kidney cancer is the ninth most common cancer in men and the fourteenth in women globally[1], characterized by rising incidence, poor survival rates, and a high proportion of asymptomatic cases, highlighting its potential as a suitable target for screening strategies[2]. The anatomical extent of kidney cancer is closely linked to prognosis, with 25% of patients already exhibiting metastasis at diagnosis[3]. Sensitive biomarkers, especially those detectable in asymptomatic individuals, could significantly improve patient outcomes[2]. Tumor development is a gradual process shaped by both genetic and environmental factors, during which biomarkers may already reflect underlying disease[4]. Understanding these preclinical biological alterations could provide valuable insights into kidney cancer pathogenesis and facilitate the identification of biomarkers for early intervention. The rise of high-throughput proteomics presents opportunities to explore these mechanisms and refine screening methods.

The human proteome, capable of assessing thousands of circulating proteins simultaneously, reflects the complex interplay of genetic and epigenetic regulation in biological systems. Proteins, as the ultimate biological effectors, offer direct insights into disease pathophysiology[5]. While earlier studies have linked individual plasma or urinary proteins to kidney cancer risk, they were limited by small sample sizes, cross-sectional designs, or short follow-up periods^[6-8]^, often overlooking proteomic changes over prolonged asymptomatic periods and potentially introducing reverse causation^[9,10]^. The UK Biobank, with its large-scale plasma protein data and long-term follow-up of over 50 000 participants, provides a unique opportunity to address these limitations and explore the molecular mechanisms of kidney cancer, paving the way for the identification of potential biomarkers.

**Figure 1.**
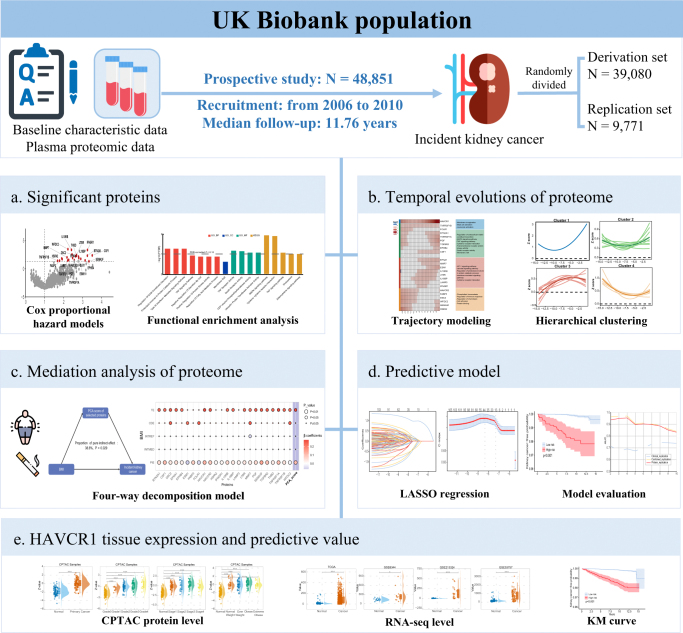
Study overview. A total of 48 851 participants with proteomic data were included, with a median follow-up of 11.76 years and 165 incident kidney cancer cases. (a) Identification of significant proteins associated with incident kidney cancer. (b) Temporal changes in the proteome up to 15 years before diagnosis. (c) Mediation analysis of proteome linking body mass index, smoking, and kidney cancer incidence. (d) Development of a proteomics-based prediction model. (e) Analysis of HAVCR1 expression and its predictive value.

In this study, we performed a proteome-wide association analysis to identify plasma proteins associated with incident kidney cancer. Temporal trajectories of these proteins were modeled up to 15 years prior to diagnosis. To explore potential mechanisms, we applied a mediation analysis using a four-component framework to assess how the plasma proteome mediates the effects of obesity and smoking on kidney cancer risk. To evaluate the predictive utility of these proteins, we constructed a risk prediction model using least absolute shrinkage and selection operator (LASSO) regression. Finally, we focused specifically on the association between pre-diagnostic plasma HAVCR1 levels and incident kidney cancer (Fig. 1a–e).

## Methods

### Study populations

This study was conducted using data from the UK Biobank, a large-scale prospective cohort comprising over 500 000 participants aged 37–73 years, recruited between 2006 and 2010 from 22 assessment centers across the UK. Baseline data, including health, genetic, and environmental information, were collected through biological samples and questionnaires[11]. Plasma proteomic data were available for a subset of participants, provided by the UK Biobank Pharma Proteomics Project (UKB-PPP). The UK Biobank was ethically approved by the North West Multi-Center Research Ethics Committee, with all participants providing written informed consent. This study has been reported in line with the STROCSS guidelines[12].

### Outcome and covariates

The outcomes of incident kidney cancer in the UK Biobank were identified through electronic health record linkages to the National Health Service central registers and death registries in England, Wales, and Scotland. Kidney cancer were defined using the International Classification of Diseases, 10th Revision (ICD-10) code C64, as recorded in national cancer registries. Of the 53 014 participants with available protein data, 3431 individuals with a history of cancer (excluding non-melanoma skin cancer, ICD-10 code C44) at baseline were excluded, leaving 49 583 participants. After excluding 732 participants with missing data on any covariates (age, sex, ethnicity, Townsend deprivation index [TDI], smoking status, alcohol consumption, body mass index (BMI), and histories of hypertension and diabetes), the final cohort consisted of 48 851 participants. Detailed information on the covariates is provided in Supplemental Digital Content, Supplementary Methods (available at: http://links.lww.com/JS9/F78).

Follow-up time was calculated from baseline to the earliest of the following events: primary kidney cancer diagnosis, death, loss to follow-up, or the last date of follow-up. The cancer registry cut-off dates were 31 December 2020, for England, 31 December 2016, for Wales, and 30 November 2021, for Scotland. The cohort was randomly divided into two groups: 80% for the derivation set, where the main analyses were conducted, and 20% for the replication set, reserved for validation. Details on participant selection are provided in Supplemental Digital Content, Figure S1 (available at: http://links.lww.com/JS9/F76), and field codes are listed in Supplemental Digital Content, Table S1 (available at: http://links.lww.com/JS9/F77).

### Blood proteomics

The UKB-PPP dataset includes over 50 000 samples, covering a wide range of systems and diseases. Detailed information regarding data generation, quality control, and processing is provided in Supplemental Digital Content, Supplementary Methods (available at: http://links.lww.com/JS9/F78). In summary, protein measurements were obtained at baseline for each participant using the Olink Proximity Extension Assay, which quantifies 2923 unique proteins across panels focused on cardiometabolic health, inflammation, neurology, and oncology. Protein expression levels were reported as Normalized Protein Expression (NPX) values[5]. The distribution of significant plasma proteins that passed false discovery rate (FDR) correction, shown in Supplemental Digital Content, Figure S2 (available at: http://links.lww.com/JS9/F76), approximates a normal distribution. Of the 2923 proteins, 12 were excluded due to more than 20% missing values, leaving 2911 proteins for further analysis. Missing data for these proteins were imputed using the k-nearest-neighbor algorithm (k = 10) from the impute R package[13].

### Statistical analyses

Baseline characteristics were compared between individuals with incident kidney cancer and healthy controls using Student’s t-test for continuous variables and the chi-square test or Fisher’s exact test for categorical variables. Cox proportional hazards models were employed to evaluate the relationship between plasma protein levels and incident kidney cancer, adjusting for confounders including age, sex, ethnicity, TDI, smoking status, alcohol consumption, BMI, and histories of hypertension and diabetes within the derivation set. The proportional hazards assumption was assessed using Schoenfeld residuals analysis with a *P*-value threshold of 0.01, alongside visual inspection of the corresponding scatter plots, revealing no violations for significant proteins after Bonferroni correction (Supplemental Digital Content, Figure S3, available at: http://links.lww.com/JS9/F76 and Supplemental Digital Content, Table S2, available at: http://links.lww.com/JS9/F77). Statistical significance was defined as a *P*-value of less than 1.7 × 10^−5^ (0.05/2911), corresponding to the Bonferroni-adjusted threshold. Sensitivity analyses were conducted by excluding cases diagnosed within 2 years of blood collection to reduce reverse causation bias. All analyses were performed using R statistical software (version 4.2.3). This work has been performed and documented following the STROCSS criteria[14].

### Functional enrichment analysis

To investigate the functional enrichment of the identified significant proteins, we employed the Enrichr platform (https://maayanlab.cloud/Enrichr/) using the full set of Olink panel proteins as the background gene set[15]. The analysis focused on Gene Ontology (GO) terms and Kyoto Encyclopedia of Genes and Genomes (KEGG) pathways. To account for multiple testing, the Benjamini–Hochberg correction was applied, with an enrichment FDR threshold set at 0.10[16].

### Trajectory modeling of plasma protein fluctuations and clustering

Since only baseline protein concentrations were available, the time from plasma collection to kidney cancer diagnosis was used as a surrogate for cancer progression. Trajectories of plasma proteins associated with incident kidney cancer were modeled as a function of follow-up years. To minimize inter-individual variability, each plasma protein NPX value was adjusted for age, sex, ethnicity, TDI, smoking status, alcohol consumption, BMI, and histories of hypertension and diabetes. Participants without kidney cancer served as the reference group, with each case matched to 10 controls by baseline age and sex. The protein levels of kidney cancer patients were converted to z-scores by subtracting the mean and dividing by the standard deviation of the matched controls^[17,18]^.

The locally estimated scatterplot smoothing (LOESS) method was used to model the relationship between each plasma protein and disease progression, measured in years leading up to kidney cancer diagnosis[18]. Plasma proteins with z-scores greater than 0.45 were considered abnormally elevated. Hierarchical clustering was then employed to categorize the predicted trajectories into four subgroups, with the optimal number of clusters determined through dendrogram inspection (Supplemental Digital Content, Figure S4, available at: http://links.lww.com/JS9/F76). Pairwise differences between LOESS predictions were quantified using the maximum distance metric, followed by hierarchical clustering with the complete linkage method^[19,20]^. To assess the stability of the clustering results, a sensitivity analysis was performed using k-means clustering and t-SNE visualization. Finally, GO and KEGG pathway analyses were conducted to annotate the functional characteristics of the identified clusters.

### Mediation analysis and principal components analysis (PCA)

Obesity and smoking are well-established risk factors for kidney cancer[21]. To investigate the mediating role of plasma proteins in the associations between obesity, smoking, and kidney cancer, we performed mediation analysis using the Stata med4way command, which decomposes the total effect (TE) into four components: controlled direct effect (CDE), reference interaction (INTref), pure indirect effect (PIE), and mediated interaction (INTmed)[22]. BMI was standardized to a mean of 0 and a standard deviation (SD) of 1, and smoking status was classified into two groups (current/ever vs. never). Mediation was considered significant if both TE and PIE had *P* values below 0.05. PCA was applied to reduce dimensionality and assess the collective mediating influence of proteins, with components extracted based on common variance[23]. The number of components was determined based on the Kaiser criterion and a scree plot (Supplemental Digital Content, Figure S5a and b, available at: http://links.lww.com/JS9/F76). Further details on the mediation analysis are provided in Supplemental Digital Content, Supplementary Methods (available at: http://links.lww.com/JS9/F78).

### Risk prediction model construction

Three risk prediction models were developed to assess kidney cancer risk. The clinical model was built using significant clinical risk factors identified via stepwise Cox regression guided by the minimum Akaike Information Criterion. The protein model included proteins selected through Cox regression with FDR correction, followed by variable refinement using LASSO regression. The combined model integrated both clinical and protein risk factors through stepwise Cox regression. Model performance was evaluated using the concordance index (C-index), area under the time-dependent ROC curve (AUC), and integrated discrimination improvement (IDI). Differences in C-index across the models were assessed using the survcomp package. Detailed methodology is provided in Supplemental Digital Content, Supplementary Methods (available at: http://links.lww.com/JS9/F78).

### Expression of HAVCR1 across different datasets

To evaluate HAVCR1 expression in tumor tissues, we analyzed protein levels from both primary cancer and adjacent normal tissues using data from the Clinical Proteomic Tumor Analysis Consortium (CPTAC) dataset. In addition, we investigated differential gene expression between tumor and normal tissues across several bulk transcriptome datasets, including The Cancer Genome Atlas (TCGA), GSE213324, GSE6344, and GSE53757. The datasets were obtained from TCGA (https://portal.gdc.cancer.gov/) and the Gene Expression Omnibus (GEO) (https://www.ncbi.nlm.nih.gov/geo/), all of which are publicly accessible.

## Results

### Population characteristics

A total of 48 851 participants without a history of cancer at enrollment were included in this study. Among these, 21 385 (43.78%) were aged 60 years or older, 45 738 (93.63%) self-reported as white, and 22 944 (46.97%) were male. During a median follow-up of 11.76 years (interquartile range [IQR] 11.04-12.50), 165 participants (0.34%) developed incident kidney cancer. Among these cases, 111 (67.3%) were aged 60 years or older, 161 (97.6%) self-reported as white, and 110 (66.7%) were male, indicating a higher incidence among males. The cohort was randomly divided into an 80% derivation set (n = 39 080) and a 20% replication set (n = 9771). Over a median follow-up of 11.76 years (IQR 11.04, 12.50) in the derivation set and 11.77 years (IQR 11.05, 12.50) in the replication set, 126 (0.32%) and 39 (0.40%) cases were observed, respectively. Supplemental Digital Content, Table S3 (available at: http://links.lww.com/JS9/F77) presents an overview of participant characteristics and differences between the derivation and replication sets.

### Associations between plasma proteins and incident kidney cancer

**Figure 2.**
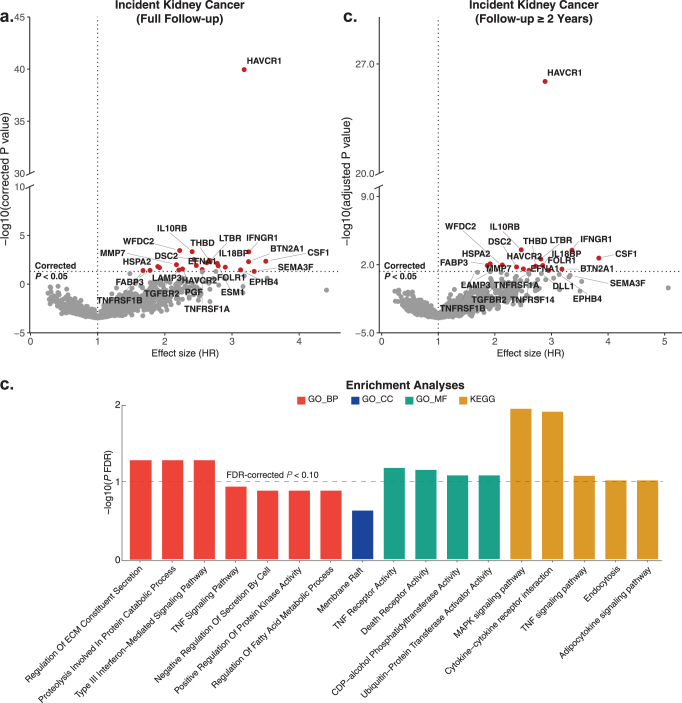
Proteins associated with incident kidney cancer and functional enrichment analysis. (a) The volcano plot illustrates proteins significantly associated with kidney cancer over the full follow-up period, identified through Cox regression analysis with a Bonferroni-corrected *P*-value threshold of <0.05. (b) Sensitivity analysis, excluding patients with less than 2 years of follow-up, further confirms significant protein associations with kidney cancer, maintaining the Bonferroni-corrected *P*-value threshold of <0.05. (c) Functional enrichment analysis was performed on proteins that surpassed the Bonferroni correction criteria, using the Enrichr platform (https://maayanlab.cloud/Enrichr/) with Olink proteins as the background gene set. Enrichment significance was set at a false discovery rate (FDR)-corrected *P* value of less than 0.10, marked by the dotted horizontal line. Abbreviations: HR, hazard ratio; GO, Gene Ontology; BP, biological process; CC, cellular component; MF, molecular function; KEGG, Kyoto Encyclopedia of Genes and Genomes; TNF, tumor necrosis factor; ECM, extracellular matrix.

Cox proportional hazards models were utilized to evaluate the associations between plasma proteins and incident kidney cancer, adjusting for age, sex, ethnicity, TDI, smoking status, alcohol consumption, BMI, and histories of hypertension and diabetes. Among the 2911 plasma proteins analyzed (Supplemental Digital Content, Table S4, available at: http://links.lww.com/JS9/F77), 24 proteins were significantly associated with kidney cancer risk after Bonferroni correction (*P* < 1.7 × 10^−5^), all of which were increased in patients with kidney cancer (Supplemental Digital Content, Figure S2a, available at: http://links.lww.com/JS9/F77 and Supplemental Digital Content, Table S5, available at: http://links.lww.com/JS9/F77). Notably, HAVCR1 demonstrated the most statistically significant association with kidney cancer risk (HR = 3.18, 95% confidence interval [CI]: 2.70–3.74, *P* = 1.11 × 10^−40^), followed by WFDC2 (HR = 2.22, 95% CI: 1.65–2.99, *P* = 3.58 × 10^−4^), IL10RB (HR = 2.41, 95% CI: 1.73–3.34, *P* = 4.66 × 10^−4^), IFNGR1 (HR = 3.25, 95% CI: 2.09–5.05, *P* = 4.86 × 10^−4^), DSC2 (HR = 2.44, 95% CI: 1.71–3.48, *P* = 3.00 × 10^−3^), and LTBR (HR = 2.68, 95% CI: 1.80–3.99, *P* = 3.93 × 10^−3^), in order of decreasing statistical significance. To reduce potential bias from reverse causation and confounding by renal function, we excluded participants diagnosed within 2 years of baseline and performed sensitivity analyses adjusting for serum creatinine. In both analyses, all 24 proteins remained significantly associated with incident kidney cancer (Supplemental Digital Content, Table S6, available at: http://links.lww.com/JS9/F77 and Supplemental Digital Content, Table S7, available at: http://links.lww.com/JS9/F77). Notably, HAVCR1 consistently demonstrated the most statistically significant association, with a hazard ratio (HR) of 2.89 (95% CI: 2.41–3.47; *P* = 1.32 × 10^−26^) after excluding early cases (Fig. 2b), and an HR of 3.29 (95% CI: 2.79–3.89; *P* = 1.34 × 10^−44^) after adjustment for creatinine.

We performed functional enrichment analysis of the 24 identified proteins to elucidate the biological mechanisms underlying kidney cancer (Supplemental Digital Content, Tables S8 and S9, available at: http://links.lww.com/JS9/F77). The most significantly enriched pathways associated with incident kidney cancer included cytokine receptor interaction, regulation of extracellular matrix constituent secretion, the MAPK signaling pathway, the tumor necrosis factor (TNF) signaling pathway, and Type III Interferon-Mediated Signaling Pathway (Fig. 2c).

### Undulations of kidney cancer-related plasma proteome through clustering protein trajectories

**Figure 3.**
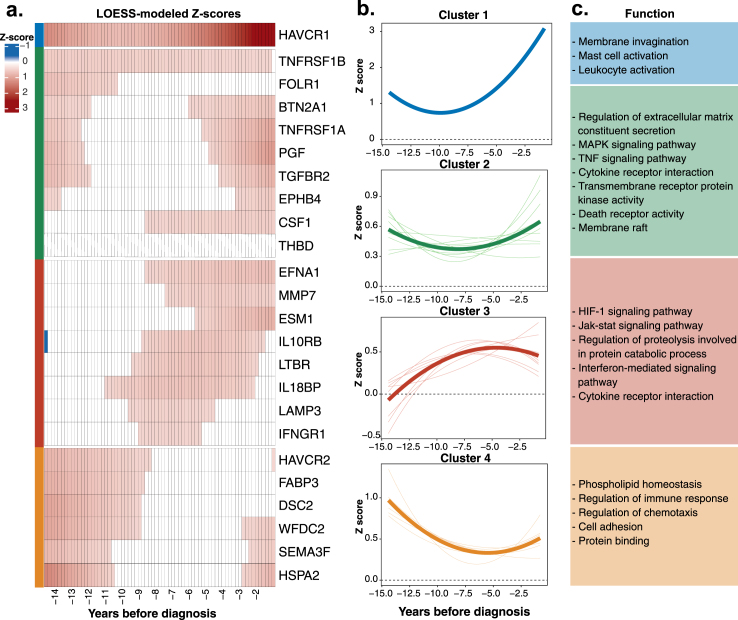
Dynamic changes in plasma protein expression preceding kidney cancer diagnosis and their trajectory clustering. (a) Longitudinal trajectories of dysregulated plasma proteins during the 15 years before kidney cancer diagnosis, modeled using the locally estimated scatterplot smoothing (LOESS) method. Protein levels were standardized as z-scores relative to matched controls. The color gradient reflects expression changes, with red indicating elevation and blue indicating reduction. (b) Proteins were grouped into four clusters based on their trajectories, identified through unsupervised hierarchical clustering. (c) Gene Ontology (GO) and Kyoto Encyclopedia of Genes and Genomes (KEGG) pathway analyses were conducted for the four distinct trajectory clusters, using the Enrichr platform (https://maayanlab.cloud/Enrichr/) with Olink proteins as the background gene set. Abbreviations: TNF, tumor necrosis factor; HIF, hypoxia-inducible factor.

To analyze and compare early fluctuations in plasma proteins preceding kidney cancer diagnosis, we modeled the trajectories of standardized protein z-scores using LOESS (Supplemental Digital Content, Table S10, available at: http://links.lww.com/JS9/F77). Figure 3a illustrates changes in 24 significant proteins. Among these proteins, HAVCR1 exhibited the most pronounced deviation, reaching abnormal levels 15 years before diagnosis and rising sharply as the disease progressed, peaking at the time of diagnosis. This highlighted HAVCR1 as a particularly promising biomarker for early detection of kidney cancer. Notably, its initial z-score exceeded 1.00, surpassing that of other proteins, suggesting that HAVCR1 levels may have begun increasing even earlier than the point of observation. While most proteins exhibited abnormal levels as diagnosis approached, their trajectories varied widely. For instance, TNFRSF1B reached early abnormality 15 years before diagnosis, with continuous fluctuations throughout the disease course. In contrast, proteins such as BTN2A1, TNFRSF1A, PGF, TGFBR2, EPHB4, WFDC2, SEMA3F, and HSPA2 displayed early abnormalities but normalized before becoming abnormal again within 6 years of diagnosis. Additionally, proteins like LTBR, IL10RB, IL18BP, LAMP3, and IFNGR1 became abnormal between 2 and 10 years before diagnosis but reverted to normal as diagnosis approached.

To identify patterns of protein trajectory variation, we performed unsupervised hierarchical clustering, which revealed four distinct protein trajectory clusters (Supplemental Digital Content, Figure S3b, available at: http://links.lww.com/JS9/F77 and Supplemental Digital Content, Table S11, available at: http://links.lww.com/JS9/F77). The stability of this classification was further confirmed by k-means clustering, which showed that only FOLR1 shifted from cluster 2 to cluster 4, while the clustering of all other proteins remained unchanged (Supplemental Digital Content, Figure S6, available at: http://links.lww.com/JS9/F76 and Supplemental Digital Content, Table S12, available at: http://links.lww.com/JS9/F77). Cluster 1 consisted solely of HAVCR1, marked by early abnormality and exponential growth. Cluster 2 included proteins with consistently high and stable z-scores over the 15 years preceding diagnosis, with many proteins becoming abnormal near diagnosis, indicating a close link to tumor development. Cluster 3 exhibited a sharp rise to abnormal levels followed by a plateau, likely reflecting near-term tumor emergence. Cluster 4 peaked around 15 years before diagnosis and then returned to normal, suggesting an association with long-term tumor progression.

Functional enrichment analysis for each cluster revealed distinct biological pathways (Supplemental Digital Content, Table S13, available at: http://links.lww.com/JS9/F77). Cluster 1, featuring HAVCR1, was associated with membrane invagination and kidney disease[24]. Cluster 2 was enriched in processes related to the regulation of extracellular matrix constituent secretion, cytokine receptor interactions, and the MAPK and TNF signaling pathways. Cluster 3 involved the HIF-1 and JAK-STAT signaling pathways, as well as cytokine receptor activity. Cluster 4 was linked to phospholipid homeostasis and cell adhesion (Fig. 3c).

### Mediation analysis

Obesity and smoking are well-established risk factors for kidney cancer. This study investigated whether the plasma proteome mediated the relationship between BMI, smoking, and kidney cancer incidence. Initially, we evaluated the associations among exposure, mediators, and outcomes using linear regression and Cox regression analyses to identify proteins that met the criteria for mediation (Supplemental Digital Content, Tables S14 and S15, available at: http://links.lww.com/JS9/F77). These proteins were further analyzed using mediation models based on a four-component framework, with adjustment for potential confounders.

**Figure 4.**
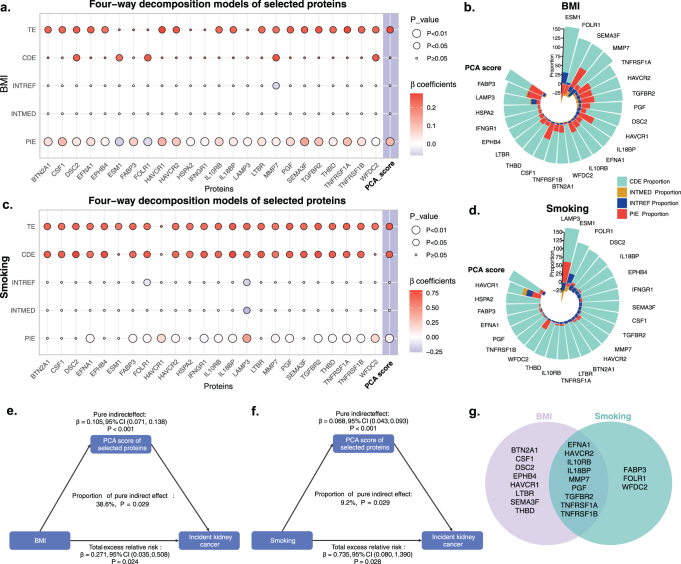
Four-component mediation analysis of the associations between BMI, smoking, and kidney cancer incidence, with mediation through plasma proteomic features. (a, b) The four-component decomposition of the BMI-kidney cancer association presented as a heatmap (a) and a circular bar plot (b). (c, d) The four-component decomposition of the smoking-kidney cancer association shown as a heatmap (c) and a circular bar plot (d). (e, f) Mediation diagrams present the mediation effects of principal component analysis (PCA) scores, representing the primary dimensions of mediation for both BMI-kidney cancer (e) and smoking-kidney cancer (f) associations. (g) A Venn diagram highlights the overlap of proteins involved in the PCA analysis, mediating both BMI-kidney cancer and smoking-kidney cancer associations. Abbreviations: TE, total effect; CDE, controlled direct effect; INTREF, reference interaction; INTMED, mediated interaction; PIE, pure indirect effect; BMI, body mass index.

In the BMI analysis, 17 of the 24 proteins exhibited a significant PIE between BMI and kidney cancer, with mediation proportions ranging from 10.6% to 57.6% (Supplemental Digital Content, Figure S4a, available at: http://links.lww.com/JS9/F77 and Supplemental Digital Content, Table S16, available at: http://links.lww.com/JS9/F77). SEMA3F emerged as the most significant mediator, accounting for 57.6% of the mediation (Fig. 4b). In the smoking analysis, 15 proteins demonstrated a significant PIE between smoking and kidney cancer, with mediation proportions ranging from 2.1% to 28.0%, excluding LAMP3 due to its significant interaction mediation effect (*P* < 0.05) (Supplemental Digital Content, Figure S4c, available at: http://links.lww.com/JS9/F77 and Supplemental Digital Content, Table S17, available at: http://links.lww.com/JS9/F77). WFDC2 was identified as the strongest mediator for smoking, with a mediation proportion of 28.0% (Fig. 4d).

To reduce dimensionality, we performed PCA on the significant mediators, excluding those with opposing signs for PIE and CDE (Supplemental Digital Content, Table S18, available at: http://links.lww.com/JS9/F77). Incorporating the PCA scores into the med4way analysis (Supplemental Digital Content, Table S19, available at: http://links.lww.com/JS9/F77), we found that the proteome explained 38.6% of BMI’s and 9.2% of smoking’s TE on kidney cancer risk (Supplemental Digital Content, Figure S4e and f, available at: http://links.lww.com/JS9/F77 and Supplemental Digital Content, Table S20, available at: http://links.lww.com/JS9/F77). These results suggested that specific plasma proteins mediated the effects of obesity and smoking on kidney cancer, with nine proteins identified as shared mediators in both pathways: EFNA1, HAVCR2, IL10RB, IL18BP, MMP7, PGF, TGFBR2, TNFRSF1A, and TNFRSF1B (Fig. 4g).

### Risk prediction model construction and evaluation

The clinical model, which included four independent risk factors – age, gender, smoking, and BMI (Supplemental Digital Content, Table S21, available at: http://links.lww.com/JS9/F77) – achieved a C-index of 0.709 (95% CI: 0.664–0.754) in the derivation set, with AUC values ranging from 0.692 to 0.742 for risk prediction of kidney cancer within 2–10 years after the initial assessment (Supplemental Digital Content, Figure S5a, available at: http://links.lww.com/JS9/F77 and Supplemental Digital Content, Table S22, available at: http://links.lww.com/JS9/F77). In the replication set, the C-index was 0.713 (95% CI: 0.623–0.803), with AUC values ranging from 0.691 to 0.774 (Supplemental Digital Content, Figure S5b, available at: http://links.lww.com/JS9/F77 and Supplemental Digital Content, Table S22, available at: http://links.lww.com/JS9/F77). For the protein model, LASSO regression was applied to the proteins selected via FDR correction, ultimately identifying 11 proteins for inclusion in the model (Supplemental Digital Content, Figure S5c and d, available at: http://links.lww.com/JS9/F77 and Supplemental Digital Content, Table S23, available at: http://links.lww.com/JS9/F77). In the derivation set, this model demonstrated superior predictive performance, with a C-index of 0.823 (95% CI: 0.782–0.865) and AUC values ranging from 0.817 to 0.949 for the 2–10 year prediction window (Supplemental Digital Content, Figure S5a, available at: http://links.lww.com/JS9/F77 and Supplemental Digital Content, Table S22, available at: http://links.lww.com/JS9/F77). In the replication set, the C-index increased to 0.811 (95% CI: 0.726–0.896), with AUC values between 0.833 and 0.954, significantly outperforming the clinical model (*P* = 0.029) (Supplemental Digital Content, Figure S5b, available at: http://links.lww.com/JS9/F77 and Supplemental Digital Content, Table S22, available at: http://links.lww.com/JS9/F77).

**Figure 5.**
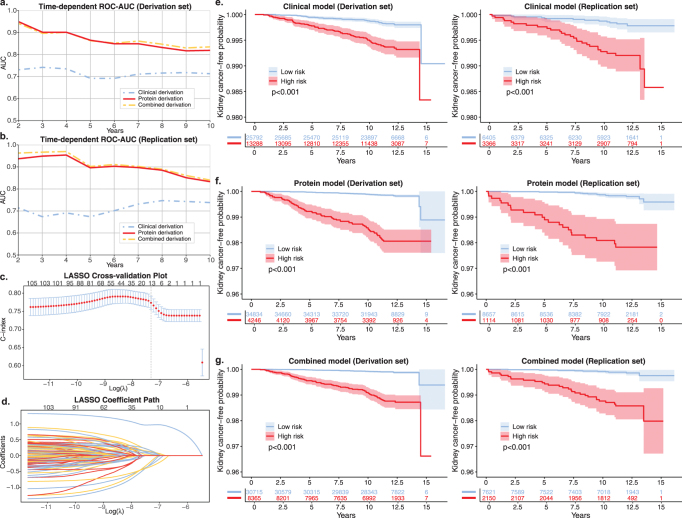
Evaluation of clinical, protein, and combined risk prediction models. (a, b) Time-dependent ROC-AUC predict the risk of incident kidney cancer over a 2–10-year period using three distinct models within both the derivation (a) and replication cohorts (b). (c, d) LASSO regression enables dimensionality reduction of proteomic data, as illustrated by the LASSO Cross-validation Plot (c) and the LASSO Coefficient Path (d). (e–g) Kidney cancer-free survival curves for the high- and low-risk groups in the clinical (e), protein (f), and combined (g) models, based on classification using the optimal cut-off value derived from the Youden index. Abbreviations: ROC-AUC, Receiver operating characteristic – area under the curve; LASSO, Least absolute shrinkage and selection operator.

By applying feature fusion to the clinical and protein features, we subsequently developed the combined model through stepwise Cox regression analysis (Supplemental Digital Content, Table S24, available at: http://links.lww.com/JS9/F77). The combined model exhibited strong predictive performance (C-index: 0.838, 95% CI: 0.798–0.878 in the derivation set; C-index: 0.812, 95% CI: 0.723–0.902 in the replication set, *P* = 0.011), though it did not show a substantial improvement over the protein model alone (Supplemental Digital Content, Figure S5a and b, available at: http://links.lww.com/JS9/F77 and Supplemental Digital Content, Table S22, available at: http://links.lww.com/JS9/F77). Nevertheless, IDI analysis revealed that both the protein model and the combined model significantly improved predictive performance compared to the clinical model, with increases of 2.9% (*P* < 0.001) and 3.9% (*P* < 0.001), respectively. Furthermore, Kaplan–Meier curves revealed that the clinical (Fig. 5e), protein (Fig. 5f), and combined models (Fig. 5g) significantly separated kidney cancer patients from non-cancer individuals during the follow-up period, with all *P* values <0.001. These findings underscore the importance of plasma proteomics in predicting incident kidney cancer, highlighting its robust predictive capability even up to 10 years prior to diagnosis.

### The role of HAVCR1 in incident kidney cancer

After Bonferroni correction, HAVCR1 emerged as the most significant plasma protein independently associated with incident kidney cancer (HR = 3.181, 95% CI: 2.704–3.744, *P* = 3.82 × 10^−44^) (Fig. 2a). LOESS analysis revealed that HAVCR1 expression increased as early as 15 years before diagnosis, with differential expression becoming more pronounced as the diagnosis approached (Fig. 3a). Furthermore, Kaplan–Meier analysis demonstrated that HAVCR1 significantly distinguished kidney cancer patients from non-cancer individuals within the overall population (*P* < 0.001) (Supplemental Digital Content, Figure S7a, available at: http://links.lww.com/JS9/F76). Cox regression models indicated a substantial predictive capacity for HAVCR1, achieving a C-index of 0.754 (95% CI: 0.709–0.799). ROC analysis further illustrated that the predictive AUC values increased from 0.770 ten years prior to diagnosis to 0.898 two years before (Supplemental Digital Content, Figure S7b, available at: http://links.lww.com/JS9/F76 and Supplemental Digital Content, Table S25, available at: http://links.lww.com/JS9/F77), aligning with trends observed in the LOESS analysis. These findings suggested that HAVCR1 possessed significant capacity for predicting incident kidney cancer, particularly concerning the likelihood of disease occurrence in the near term.

**Figure 6.**
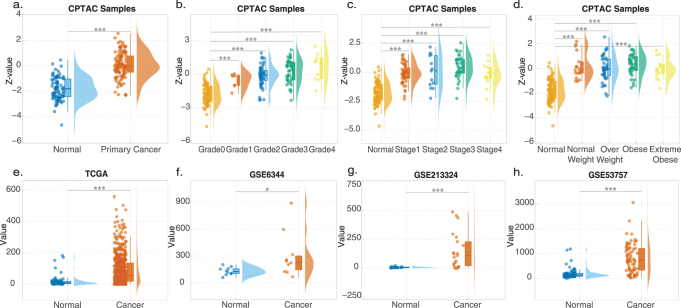
Tissue expression of HAVCR1 in kidney cancer. (a–d) Using CPTAC data, comparative analysis reveals HAVCR1 protein expression levels in tumor versus adjacent normal tissues (a), and its distribution across tumor grades (b), stages (c), and obesity status (d). (e–h) Differential HAVCR1 RNA expression between tumor and adjacent normal tissues is validated by TCGA (e), GSE6344 (f), GSE213324 (g), and GSE53757 (h) datasets. *P < 0.05, **P < 0.01, ***P < 0.001. Abbreviations: ROC-AUC, receiver operating characteristic – area under the curve; CPTAC, Clinical Proteomic Tumor Analysis Consortium.

To deepen our understanding of the relationship between HAVCR1 and kidney cancer, we analyzed protein data from tumor patients in the CPTAC database, revealing significantly higher HAVCR1 expression in tumor tissues compared to adjacent normal tissues (Fig. 6a). Elevated HAVCR1 expression was associated with higher tumor grade (Fig. 6b), advanced stage (Fig. 6c), and obesity (Fig. 6d). In RNA-level analyses, HAVCR1 expression was significantly elevated in tumors relative to adjacent normal tissues, as confirmed by the TCGA (Fig. 6e), GSE6344 (Fig. 6f), GSE213324 (Fig. 6g), and GSE53757 (Fig. 6h) datasets, underscoring its strong association with cancer development.

## Discussion

This study represents the largest prospective proteomic analysis to date, investigating plasma proteins associated with incident kidney cancer risk in a natural population over 15 years prior to diagnosis. We identified 24 proteins significantly linked to kidney cancer risk, providing novel insights into their roles in disease development. HAVCR1 emerged as the most critical marker, showing marked fluctuations long before diagnosis and demonstrating strong predictive performance (C-index = 0.754). The discovery of four distinct temporal protein variation patterns further underscored the possibility that different proteins influenced kidney cancer risk at specific stages of disease progression. Moreover, our findings revealed the potential mediating role of these proteins in the relationships between BMI, smoking and kidney cancer risk, explaining 38.6% and 9.2% of the TE, respectively. The proteomic risk model, with a C-index of 0.811, emphasized the potential of plasma protein profiles for identifying high-risk individuals for early detection and prevention.

Proteins, as the final products of gene expression and essential mediators of cellular functions, play a crucial role in disease progression, positioning them as promising candidates for biomarker discovery[25]. Recent advances in high-throughput proteomics have enhanced our understanding of various diseases, particularly in improving the prediction of major cardiovascular events and the accuracy of clinical models for chronic kidney disease^[26,27]^. Proteomic analyses have also shown promise in early cancer detection, such as lung, prostate, hepatocellular carcinoma, and multiple myeloma, underlining its significance in cancer risk assessment^[28–31]^. Building on these advancements, Papier *et al* performed an extensive analysis of UK Biobank proteomic data, profiling 1463 proteins across 19 cancer types. Their study identified 371 proteins associated with the risk of at least one cancer, with 146 linked to multiple cancer types, suggesting shared tumorigenic pathways[4]. They observed that protein-cancer associations were particularly strong in hematological malignancies and highly vascularized cancers, including kidney, liver, and lung cancers[4]. In our study, using over 2900 plasma protein datasets from the 2023 UK Biobank release[5], we identified 24 proteins significantly associated with kidney cancer risk. Consistent with previous research, we observed elevated levels of HAVCR1, MMP7, and ESM1 in kidney cancer patients^[10,32,33]^. Specifically, HAVCR1 is implicated in renal tubule injury[34], MMP7 in extracellular matrix degradation[34], and ESM1 in angiogenesis[35]. Although ANGPTL4 and SPP1 have been proposed as potential serum biomarkers for kidney cancer^[34,36]^, they did not reach Bonferroni significance in our analysis of incident disease risk, despite passing the FDR threshold.

This suggested the need for further validation of their predictive value. Our data-driven proteomics approach identified novel serum proteins associated with kidney cancer, emphasizing their potential as early detection biomarkers.

This study investigated the longitudinal trajectory of kidney cancer-associated proteins over the 15 years preceding diagnosis, filling a critical gap in understanding the disease’s natural progression. Notably, HAVCR1 exhibited the most significant fluctuations, with levels persistently rising and peaking at diagnosis. A prospective nested case-control study also reported that plasma HAVCR1 levels could predict kidney cancer up to 5 years before diagnosis[7]. However, previous research had not explored the temporal dynamics of HAVCR1 in relation to kidney cancer development^[7,8,37]^. LOESS analysis revealed distinct nonlinear patterns in protein activity, indicating their potential as early markers of tumor initiation. Cluster 2, characterized by persistent dysregulation, points to a prolonged period of cellular vulnerability before kidney cancer onset, offering valuable insights for screening high-risk populations and early intervention. This cluster was notably associated with cytokine receptor interactions and the MAPK signaling pathway, both of which are integral to the growth and angiogenesis of renal cell carcinoma[38]. In contrast, Cluster 3 was closely linked to the near-term onset of tumors, indicating an accelerated stage of tumor development. These findings provide important signals for predicting the near-term onset of tumors, as proteins in this cluster may facilitate cancer progression through the HIF-1 and JAK-STAT signaling pathways^[39,40]^. Conversely, Cluster 4, associated with long-term tumor progression, displayed early deviations in proteins related to phospholipid homeostasis and cell adhesion, followed by a gradual decline. Recognizing these distinct phases of protein activity deepens our understanding of kidney cancer pathogenesis and offers valuable insights for developing future early detection strategies.

Using a mediation analysis based on a four-component framework, this study evaluated the role of proteomic features in mediating the associations between kidney cancer incidence and established risk factors, namely obesity and smoking. While the associations between these factors and kidney cancer are well-established, the underlying biological mechanisms remain unclear^[41–43]^. We identified 17 proteins that mediated the connection between BMI and kidney cancer, many implicated in obesity^[44–48]^. For instance, TNFRSF1A, linked to obesity[48], significantly impacted the proliferation and migration of kidney cancer cells[49]. Similarly, PGF, which promotes angiogenesis and endothelial cell growth, was elevated in obese individuals and associated with tumor progression^[46,50]^. Our PCA integration of proteomic data revealed that proteins mediated 38.6% of the relationship between BMI and kidney cancer incidence. This finding suggests that the mediating proteins offer insights into the underlying mechanisms while also providing potential therapeutic value. Smoking nearly doubled the risk of kidney cancer[41]. We identified 15 proteins that mediated the relationship between smoking and kidney cancer, many linked to smoking exposure^[51–55]^. Notably, WFDC2, a pro-fibrotic proteinase inhibitor involved in innate immunity, was overexpressed in various cancers and associated with smoking[56]. MMP7, known for its role in tissue remodeling, was also significantly linked to smoking and influenced kidney cancer development and prognosis[32]. Our PCA analysis determined that plasma proteins mediated 9.2% of the association between smoking and kidney cancer. Interestingly, nine proteins were identified as common mediators in both the BMI-kidney cancer and smoking-kidney cancer pathways, suggesting shared biological mechanisms by which these risk factors contribute to kidney cancer development.

HAVCR1, a transmembrane glycoprotein shed by kidney cancer cells and detectable in both blood and urine, is among the most extensively studied biomarkers for kidney cancer. Several small-scale retrospective analyses reported elevated urinary HAVCR1 levels in patients compared to healthy controls, supporting its diagnostic utility^[9,37,57]^. However, these studies mainly relied on cross-sectional data from limited cohorts and lacked longitudinal insight. Here, we present the first large-scale prospective evaluation of plasma HAVCR1 in relation to kidney cancer risk. Using LOESS-based trajectory analysis, we revealed a gradual increase in HAVCR1 levels starting up to 15 years before diagnosis, with acceleration near clinical onset. This notably extends the early detection window beyond the 5 years reported by Scelo *et al*[7], whose nested case–control design had limited temporal resolution. Moreover, while previous studies showed that pre-nephrectomy plasma HAVCR1 can distinguish malignant from benign renal masses and predict prognosis^[8,58]^, those findings were mostly from symptomatic or radiologically selected cohorts. Our population-based analysis of asymptomatic individuals prior to clinical manifestation highlights HAVCR1’s potential for early risk stratification at the population level. Consistent with prior tissue analyses[58], we confirmed significantly higher HAVCR1 expression in tumor versus adjacent non-tumor tissues, correlating with tumor stage and grade. Importantly, integrating HAVCR1 into the proteomics-driven risk model improved its discriminative performance, emphasizing its value within a comprehensive early detection framework for kidney cancer.

Early detection is crucial for improving outcomes in kidney cancer, particularly in its localized stages, which often progress silently without obvious symptoms. However, as highlighted by the experience with prostate-specific antigen testing in prostate cancer, screening strategies must carefully weigh the benefits of early diagnosis against the risks of overdiagnosis[59]. Recent advances in multi-omics technologies, especially proteomics, have opened new avenues for identifying reliable biomarkers for early risk assessment[60]. In this study, we developed a plasma proteomics–based risk model that predicts incident kidney cancer with high discrimination (C-index = 0.811). The model generates a continuous risk score that facilitates individualized risk stratification, enabling more tailored surveillance – for instance, recommending more frequent imaging or lifestyle interventions for those at higher risk. Notably, the model maintains stable predictive performance over time, with time-dependent AUCs ranging from 0.833 to 0.954 across 2–10 years of follow-up. This temporal consistency enables dynamic risk stratification, allowing clinicians to tailor surveillance intensity to short-, intermediate-, and long-term risk profiles. Patients with higher short-term risk may benefit from earlier or more frequent imaging, while lower-risk individuals could undergo less intensive monitoring. Moreover, the plasma proteomic assay is non-invasive, cost-effective, and easy to implement, making it well-suited for incorporation into routine health evaluations or broader population-level screening. It may also aid in interpreting ambiguous imaging findings, improving diagnostic confidence and reducing unnecessary interventions^[8,61]^. Beyond kidney cancer, plasma proteomics enables simultaneous risk evaluation for multiple diseases, thereby improving screening efficiency and cost-effectiveness. Despite these advances, identifying the optimal approach to early kidney cancer screening remains a complex and evolving challenge.

This study is the first integration of high-throughput proteomic profiling with detailed assessments of incident kidney cancer in a large cohort with long-term clinical follow-up, offering insights into dynamic changes of kidney cancer-related proteins prior to disease. However, several limitations should be considered. First, the study population largely consisted of individuals of European descent, requiring further resear2ch on the applicability of the results to other ethnic groups. Second, the relatively low incidence of kidney cancer limited the number of cases within the UK Biobank, so outcomes were defined using the ICD-10 code C64[4], without subtype-specific analyses. Third, while Olink technology enabled extensive measurement of plasma proteins, it does not capture the full spectrum, potentially overlooking key proteins associated with cancer risk. Additionally, this method may introduce bias toward certain protein subsets. Fourth, the absence of tumor grading and staging data in the UK Biobank limits the application of plasma proteomics to specific kidney cancer types. Public datasets (CPTAC, TCGA, and GEO) show that HAVCR1 expression in tumor tissue correlates with higher tumor grade and stage, highlighting the potential value of exploring the longitudinal relationship between plasma proteomics and tumor characteristics.

## Conclusion

In conclusion, this prospective study represents the largest plasma proteome analysis in kidney cancer, identifying biomarkers associated with disease onset, with HAVCR1 emerging as a promising biomarker. For the first time, we uncovered the temporal evolution of different proteins prior to kidney cancer diagnosis, revealing key pathological changes throughout disease trajectory. Furthermore, our findings clarified the mediating role of proteomics in the interaction between obesity, smoking, and incident kidney cancer. These findings fill important knowledge gaps concerning the pathophysiological precursors of kidney cancer and provide a foundation for developing early screening strategies.

## Data Availability

Data utilized in this study are accessible from several sources. UK Biobank data are available through the UK Biobank website (https://www.ukbiobank.ac.uk/) under project number 184070. Gene expression RNA-seq and clinical data from TCGA can be obtained via the GDC portal (https://portal.gdc.cancer.gov/), while additional gene expression datasets are accessible through the GEO platform (GSE213324, GSE6344, and GSE53757) (https://www.ncbi.nlm.nih.gov/geo/). Protein data from CPTAC are available for analysis through the UALCAN portal (https://ualcan.path.uab.edu/cgi-bin/ualcan-res-prot.pl).

## Research registration unique identifying number (UIN)

Not available.
